# Revisiting Gaussian Process Regression Modeling for Localization in Wireless Sensor Networks

**DOI:** 10.3390/s150922587

**Published:** 2015-09-08

**Authors:** Philipp Richter, Manuel Toledano-Ayala

**Affiliations:** Facultad de Ingeniería, Universidad Autónoma de Querétaro, Cerro de las Campanas s/n., Col. Las Campanas, Santiago de Querétaro 76010, Mexico; E-Mail: toledano@uaq.mx

**Keywords:** sensor modeling, WLAN received signal strength, Gaussian process regression, machine learning, location fingerprinting

## Abstract

Signal strength-based positioning in wireless sensor networks is a key technology for seamless, ubiquitous localization, especially in areas where Global Navigation Satellite System (GNSS) signals propagate poorly. To enable wireless local area network (WLAN) location fingerprinting in larger areas while maintaining accuracy, methods to reduce the effort of radio map creation must be consolidated and automatized. Gaussian process regression has been applied to overcome this issue, also with auspicious results, but the fit of the model was never thoroughly assessed. Instead, most studies trained a readily available model, relying on the zero mean and squared exponential covariance function, without further scrutinization. This paper studies the Gaussian process regression model selection for WLAN fingerprinting in indoor and outdoor environments. We train several models for indoor/outdoor- and combined areas; we evaluate them quantitatively and compare them by means of adequate model measures, hence assessing the fit of these models directly. To illuminate the quality of the model fit, the residuals of the proposed model are investigated, as well. Comparative experiments on the positioning performance verify and conclude the model selection. In this way, we show that the standard model is not the most appropriate, discuss alternatives and present our best candidate.

## 1. Introduction

The knowledge of location is crucial for an uncountable number of applications driven by an ever-increasing number of mobile users. The Global Navigation Satellite System (GNSS) covers most of these applications very well; however, in dense urban areas and indoor environments, tracking of GNSS signals is unreliable due to signal attenuation and blocking, thus creating a demand for aiding and alternative positioning systems. Localization in wireless sensor networks presents such an alternative, whereupon a wireless local area network (WLANs) and, in particular, WLAN-based location fingerprinting, is one of the most promising methods, because it does not need a line-of-sight to the transmitter and works with low-cost, standardized hardware. Moreover, WLAN signals penetrate walls and, hence, in contrast to GNSS, enable seamless indoor/outdoor positioning.

WLAN fingerprinting constitutes the drawback of creating the reference database, consisting of received signal strengths (RSS) at known positions: the radio map. The number of reference points (fingerprints) affects its accuracy, and establishing an elaborate prepared radio map is very laborious. The denser the fingerprints and larger the area of interest, the more expensive is this undertaking. (The smaller the distance between two signal strength measurements, the smaller the difference between the RSSs. It is worth noting that due to the quantization of RSS to one decibel, the RSSs in a small area are certainly equal. This, in turn, leads to a saturation of the accuracy improvement when densifying the fingerprint further.)

A reduction of the labor of radio map creation is widely desired [1,2]. Interpolating or predicting the signal strength allows one to reduce the number of fingerprints while retaining the positioning accuracy. Elnahrawy *et al.* [3] proposed Delaunay triangulation to interpolate between reference points, whereas Chai *et al.* [4] interpolated linearly between neighboring likelihood functions, and Howard *et al.* [5] used interpolation filters. These interpolation schemes do not capture the random nature of RSS sufficiently, in particular if the fingerprints are sparse.

The authors of [6,7,8,9] used Gaussian process regression in the context of WLAN location fingerprinting to model RSSs. Gaussian process regression is able to predict the nonlinear, spatial relation of signal strength, resulting in a continuous spatial description of RSSs. Furthermore, the computer vision community applies Gaussian process regression for localization tasks, especially in robotic applications [10,11]. Instead of RSS, spatially-unique features are extracted from camera images and stored with their corresponding location to establish reference locations. During position estimation, this process is repeated, and the resulting features are matched with the previously-stored features in which the best match corresponds to the most likely location.

In [6,7,8,9,10,11], the “default” Gaussian process model, with zero mean and squared exponential kernel, was chosen. Gaussian process models that employ the zero mean function converge to zero in areas without training data; a property that is clearly not appropriate for RSSs. Hence, the authors of [6,8] approximated the RSS decay with a linear model and estimated in an additional step its parameters. This procedure is either not realistic in areas where the access point positions are not known or would require a survey of these positions. In addition, the choice of the squared exponential kernel in these studies is unsound.

Only Bekkali *et al.* [12] investigated several Gaussian process models in order to find the most appropriate model for WLAN location fingerprinting; however, their focus was solely on indoor environments, and the results were obtained under idealized conditions. They based their study on ray-launching, a method that simulates wave propagation based on geometrical optics (reflection, diffraction, wave guiding effects, *etc.*). Though, ray-launching only considers spatial variations of the signal power, it does not simulate spatiotemporal variations, for example due to moving people and objects; the complex power patterns that occur in real-world environments are not fully captured. Furthermore, the discrete ray increment with which each ray is launched from the transmitter is known to be disadvantageous, since not all wedges might be hit by a ray, and hence, it omits possible new sources of interferences. Besides, they assessed the models only in terms of the position accuracy and did not evaluate the quality of the model fit.

An alternative method to model WLAN power spatially was recently presented by Depatla *et al.* [13]. They use WLAN power readings and approximations to Maxwell’s equations to obtain a highly accurate model of the electric field in a small area. This WLAN signal strength model is used to solve the inverse scattering problem and allows high-resolution imaging of, possible occluded, objects. Their model of the electric field can potentially be used for localization and replace the Gaussian process regression approach; however, it is even more expensive regarding labor, and its practicality for WLAN fingerprinting is doubtful.

In vision-based localization, the choice of appropriate features is a problem on its own, and the features extracted from images are diverse among different studies. Hence, each feature may require a different Gaussian process prior distribution, whose examination is not in the scope of this study.

Very accurate Gaussian process models are fundamental to achieve WLAN location fingerprinting-based seamless indoor/outdoor positioning; even more if the objective is to fuse RSS with other sensor data. Therefore, building upon [12], these models need to be re-studied in realistic conditions, with actual RSS measurements. This work revisits Gaussian process model selection for the interpolation of radio maps. It presents detailed experimental results that challenge the model choice made in former studies, analyzes different Gaussian process models with respect to indoor *and* outdoor environments and explores the most appropriate Gaussian process model. We hypothesized whether different Gaussian process regression models for indoor and outdoor environments could be obtained and if this improves the radio map accuracy compared to a common model. The fit of the Gaussian process models is evaluated by the models’ test error and the Bayesian information criterion (both obtained via cross-validation) and supplemented with an analysis of the residuals. The validity of these findings is sought by comparing the models’ localization accuracy, assessed by Gaussian process-based maximum likelihood estimators.

## 2. Gaussian Process Regression Model for WLAN RSS Fingerprinting

Interpolating WLAN RSS, to improve radio maps, equates to predicting the spatial distribution of signal strength. Such a prediction demands a description that relates coordinates in space and the corresponding sets of RSSs, received from WLAN access points. Gaussian process regression is a tool widely used in geostatistics, where it is known as Bayesian kriging, but it also drew, especially in the last decade, much attention in the machine learning community. Gaussian process regression, as a supervised learning task, permits predicting RSSs at arbitrary coordinates on the basis of acquired data.

A Gaussian process is a collection of random variables {f(x)∣x∈X} that underlie the condition that any finite subset of realizations of the process $f={\left\{f\left(x_{i}\right)\right\}}_{i=1}^{n}$ is jointly Gaussian distributed [14]. Such a process is a generalization of a normal multivariate distribution into infinite dimension: it describes a distribution over functions. A Gaussian process is fully specified by its mean function μ(x)=E[f(x)] and covariance function $\mathrm{cov}\left(f\left(x\right),f\left(x^{\prime }\right)\right)=E\left[\left(f\left(x\right)-\mu \left(x\right)\right)\left(f\left(x^{\prime }\right)-\mu \left(x^{\prime }\right)\right)\right]$. The set X is the index set, a set of possible input values.

We use a Gaussian process to model the relationship between RSS, *s*, and points in two-dimensional space, p, some subset of $R^{2}$; this spatial process is denoted by:

$$s_{i}=f\left(p_{i}\right)+\epsilon_{i}$$

The regression problem consists of interfering the non-linear function f(·) from noisy observations that emerged from an identical independent distribution and have the variance $\sigma_{\epsilon }^{2}$. When generating the radio map, one draws samples from this process, obtaining ${\left\{p_{i},s_{i}\right\}}_{i=1}^{n}$, the so-called training data. For notational convenience, we stack the training data together, yielding a 2×n matrix of input values *P* and a *n*-dimensional vector s, the target values.

From a Bayesian point of view, the interference of f(p) corresponds to estimating the posterior distribution:

(1) $$p\left(f\left(p\right)|s,P\right)=\frac{p(s|f(p),P)p(f(p))}{p(s|P)}$$

The term p(s∣f(p),P), known as the likelihood function, denotes the probability of obtaining the RSS, s, given the function f(p), where p(f(p)) presents the prior distribution of the latent function, a Gaussian process in itself.

To interpolate RSSs at arbitrary positions, Gaussian process regression exploits the covariance of neighboring RSSs. This dependence is expressed by the process’ covariance function that specifies the similarity of two function values evaluated at their input values. Assumptions on the problem or prior knowledge about it can be encoded in the mean and covariance function of the prior distribution, thus giving the model characteristics, such as different signal or noise levels, periodicity or linearity. Gaussian process’ covariance functions are based on kernel functions; functions that peak when the distance in input space is minimal and decrease with increased distance of the input points, expressing the drop-off of dependence between RSSs farther from each other. The specific choice of a covariance function depends on the problem; the most common kernel, the squared exponential kernel, is given as an example:

$$\mathrm{cov}\left(f\left(p_{i}\right),f\left(p_{j}\right)\right)=k\left(p_{i},p_{j}\right)=\exp{(-\frac{1}{2}∥p_{i}-p_{j}∥}^{2})$$

For all pairs of training targets, we denote the covariance matrix by K(P,P). Knowing that RSS observations are noisy, we encode that characteristic in the model and write the covariance function of the prior distribution as $\mathrm{cov}\left(s\right)=K\left(P,P\right)+\sigma_{\epsilon }^{2}I$, with *I* being the identity matrix. Assuming a noise that is identical independent normal distributed allows an analytic description of Gaussian process regression, as presented as follows.

The target values of the training data, s, are correlated between each other, as they are also correlated with any arbitrary target values. This fact is employed to predict RSSs, $s^{*}\equiv f\left(P^{*}\right)$. We denote the matrix of the corresponding $n^{*}$ positions by $P^{*}$ and refer to the sought target values and their index set as test data. We can describe the joint Gaussian distribution of target values, assuming a zero mean prior distribution, as:

$$\left[\begin{array}{c} s\\ s^{*} \end{array}\right]∼N\left(0,\left[\begin{array}{cc} K\left(P,P\right)+\sigma_{\epsilon }^{2}I & K(P,P^{*})\\ K(P^{*},P) & K(P^{*},P^{*}) \end{array}\right]\right)$$

where $K(P,P^{*})$ is a $n\times n^{*}$ matrix containing the covariances between all pairs of s and $s^{*}$. Conditioning this distribution on the training RSS yields again a Gaussian process with mean Function (2) and covariance Function (3):

$$\begin{array}{ccc} s^{*}|s,P,P^{*} & ∼N\left({\bar{s}}^{*},\mathrm{cov}\left(s^{*}\right)\right) & \\ {\bar{s}}^{*} & =K\left(P^{*},P\right){\left[K\left(P,P\right)+\sigma_{\epsilon }^{2}I\right]}^{-1}s & \text{ (2)}\\ \mathrm{cov}(s^{*}) & =K\left(P^{*},P^{*}\right)-K\left(P^{*},P\right){\left[K\left(P,P\right)+\sigma_{\epsilon }^{2}I\right]}^{-1}K\left(P,P^{*}\right) & \text{ (3)} \end{array}$$

This is the posterior distribution, also called predictive distribution. Note that Gaussian process regression also estimates the uncertainty of the predictions, given by the covariance function.

The principle structure of the model is determined by the mean and covariance functions, but to fit the observations appropriately, the function’s optimal hyperparameters need to be found. This is usually done by maximizing the marginal likelihood function (This log-likelihood function is usually attributed the term marginal, since it is obtained by integrating out the unobtained observations.), the probability of the data given the model, with respect to the hyperparameters. For Gaussian distributed noise, its logarithmic form is given by:

(4) $$\log p\left(s|X,\theta \right)=-\frac{1}{2}s^{T}{\left[K\left(P,P\right)+\sigma_{\epsilon }^{2}I\right]}^{-1}s-\frac{1}{2}\log\left|K\left(P,P\right)+\sigma_{\epsilon }^{2}I\right|-\frac{n}{2}\log2\pi$$

where ***θ*** collects the model’s hyperparameters and *T* denotes the matrix transpose. This function is intrinsic to Gaussian process regression, and it balances between model complexity and data fit, hence avoiding overly complex models.

The Gaussian process regression framework can also account for non-Gaussian observations. The approach to that is to replace the noise term of the covariance function ($\sigma_{\epsilon }^{2}I$) by an appropriate covariance matrix of the non-Gaussian errors. (The covariance matrix of the observation noise must be full rank.) Of course, the likelihood function becomes non-Gaussian, and approximations to the intractable integrals are required.

The above derivation uses the default mean function of zero, that is the Gaussian process converges to zero in the absence of training data. However, nonzero mean functions are possible; they provide the Gaussian process with a global structure, for instance a linear or polynomial behavior. A nonzero mean function μ(P) can be integrated into the Gaussian process regression framework by scaling Equation (2) as follows:

$${\bar{s}}^{*}=\mu \left(P^{*}\right)+K\left(P^{*},P\right){\left[K\left(P,P\right)+\sigma_{\epsilon }^{2}I\right]}^{-1}\left(s-\mu \left(P\right)\right)$$

One transforms the observations, such that the zero mean assumption holds, meaning one basically subtracts the nonzero prior mean values from the observations. After inferring the posterior distribution, the nonzero mean is added back to the predictive mean function. This works for mean functions with known parameters. Notice that the mean function of the data is not necessarily the mean function of the process.

It is also possible to make the mean function variable, in which the principle idea is the same as described above. A parametrization can be achieved by selecting a set of fix basis functions and inferring their parameters from the data. Rassmussen *et al.* [14] describe this procedure for normal distributed noise, in which case the inference of these parameters can be described analytically. This procedure has the advantage of including the uncertainty of the mean function’s parameters in the covariance function. The simplest mean function is a constant mean function, possessing a single hyperparameter.

For more details on inference with Gaussian processes, such as examples of covariance functions, we refer the reader to [14].

### On the Gaussianity of WLAN RSS

In all cited studies where Gaussian process regression was applied to predict WLAN RSS, Gaussian measurement noise was assumed (as in the previous section). The assumption of normal distributed noise facilitates regression with a Gaussian process greatly, because it allows one to describe the Bayesian interference in closed form. Even though normal distributed noise is usually assumed for WLAN RSS, this does not necessarily mean it is correct. Note that Gaussian processes regression can be used with different noise distributions, but it requires a different likelihood function that, in turn, entails cumbersome interference methods to compute the predictive distribution. In this section, we intend to justify the Gaussian process model approach with the Gaussian likelihood function for WLAN RSS and to point out the conditions for which this assumption holds.

A general valid, analytic model describing the variability of RSSs in indoor environments has not been consolidated. The distance signal power relationship is determined by the log-distance path loss model, describing the average attenuation of signal power. Random variations due to the environment, known as large-scale shadowing, have been integrated into that model by adding a normal distributed noise term (when measured in logarithmic scale) [15]. Therefore, the most common model that describes the randomness of RSS is a log-normal distribution, but this model is still not sufficiently accurate and reliable to describe RSSs. Normal distributions of RSSs have been observed under certain circumstances, but as a generally valid model, it has been refuted [16,17]. Nonetheless, several authors model RSSs with a normal distribution as a trade-off of complexity and feasibility [16,18,19,20].

To capture the complete RSS characteristic at a WLAN reference point, the RSS histogram should be recorded [20,21]. Such a comprehensive approximation of a RSS distribution can only be obtained if enough samples are observed [17,22], which requires a certain time period. Though, for most positioning applications, this is a temporal constraint that is hardly practical. In opposition to capturing a complete histogram, a single RSS sample is clearly not robust enough. Thus, a common approach is to use an average value and the variance (hence, implying normality) to capture the RSS characteristic at a reference point, even though this disregards potentially useful information.

An in-depth analysis of the statistical properties of RSSs in indoor environments can be found in [16]. These properties also facilitate interpretations and conclusions about the distributions of the regression model residuals. On this account, we extend study [16] slightly by an analysis of the distributions of averaged RSSs. The corresponding results can be found in Section 5.1, preceding the Gaussian process modeling.

## 3. Location Estimation Based on Gaussian Process Regression Models

In the previous section, we explained how RSS can be modeled spatially. This sections treats the use of these models for localization. For this study, we use a maximum likelihood estimator (MLE) to estimate a position, a standard method for parameter estimation. The sought parameter in our application is the most probable position given the measured RSSs. The maximum likelihood method considers the position p as an unknown constant, whereas the RSS observations are random variables. The function p(s∣p) expresses the likelihood that the observed RSS, *s*, was measured at position p. As the likelihood is rather a function of p, one often writes L(p). Maximizing this function with respect to p yields the ML position estimate:

(5) $$\hat{p}=\arg\max_{p}L\left(p\right)$$

Let us extend this method to fingerprinting, which incorporates RSS from n=1,…,N access points. As opposed to standard fingerprinting, which consists of recognizing a vector of RSS observations within the entries of the radio map, this probabilistic approach uses the interpolated radio map given by the predicted mean, ${\bar{s}}^{*\;n}$ (and covariance function ${\mathrm{cov}}^{n}\left(s^{*}\right)$), for the *n*-th access point. Continuing with the assumption of normal distributed RSS noise, Equations (2) and (3) provide the mean value and variance for this distribution. If we assign the same RSS observation, $s^{n}$, emitted by the *n*-th access point, repeatedly to each of the test positions $p\in P^{*}$ and collect them in the vector $s^{*,n}$, we can compute the likelihood value of receiving $s^{n}$ at all test positions $P^{*}$. We obtain a spatial, multivariate Gaussian likelihood function:

$$p\left(s^{*,n}|s^{n},P^{n},P^{*}\right)=\frac{1}{{\left(2\pi \right)}^{|P^{*}|/2}{\mathrm{cov}}^{n}{\left(s^{*}\right)}^{1/2}}\exp\left(-\frac{1}{2}{\left(s^{*,n}-{\bar{s}}^{*,n}\right)}^{T}{\mathrm{cov}}^{n}{\left(s^{*}\right)}^{-1}\left(s^{*,n}-{\bar{s}}^{*,n}\right)\right)$$

where $P^{n}$ are the training positions that correspond to the *n*-th access point and $P^{*}$ are the test positions of the Gaussian process regression.

Stressing the common assumption that RSSs from different access points are mutual independent, the joint likelihood function becomes:

(6) $$L\left(p\right)=\prod_{l=1}^{L}p\left(s^{*,n}|s^{n},P^{n},P^{*}\right)$$

Substituting the former equation into Equation (5) provides the most probable position.

Since the test positions can be arbitrarily chosen on $R^{2}$, the likelihood function is continuous. A likelihood function, as given by Equation (6), can be easily integrated into the Bayesian filtering framework. The use of that framework is two-fold: facilitating sensor data fusion and integration of a motion model.

The likelihood function can be combined with likelihood functions of other sensor readings, thus integrating RSSs with other sensor data. The extra information of a further sensor may improve the position accuracy, especially when fused with sensory data that constitutes complementary errors; for instance, data of systems that deduce a position estimate of signal propagation delays, Global Navigation Satellite Systems, among others.

The Bayesian filter additionally facilitates the integration of a motion model. A carefully-chosen motion model captures the kinematics of the moving object and is able to reduce the position error of the estimates by confining or excluding measurement outliers that are inconclusive with the prediction based on the motion model.

## 4. Experimental Setup

For this work, two independent experiments were conducted: an experiment to verify the practicability of modeling the uncertainty of RSS with Gaussian distributions; and an experiment to create RSS radio maps to be used as training data, to generate the Gaussian process models in order to predict the RSSs and to investigate the model fit. Previous to this, the equipment and tools used are specified.

To record WLAN RSS, a Unix-based laptop and an USB WLAN adapter were used. The wireless network adapter is equipped with an Atheros AR9271 chip set and a dipole antenna providing 4 dBi of additional gain. We used the API of the netlink protocol library (libnl, http://www.infradead.org/~tgr/libnl/) and the packet capture library (TCPDUMP & libpcap, http://www.tcpdump.org/) to read WLAN packages unprocessed, at the rate an access point sends packages (Only broadcast packages of access points were captured, since they contain the needed information (RSS, MAC address), and this helps to avoid concerns with respect to privacy. WLAN broadcast packages are usually sent at a rate of 100 ms). We stored the data in SQLite (http://www.sqlite.org/) databases. The positions of the fingerprints were retrieved from OpenStreetMap (https://www.openstreetmap.org) data, wherefore we integrated the package capture software into JMapViewer (http://wiki.openstreetmap.org/wiki/JMapViewer).

### 4.1. Static RSSs Measurement from a Single Access Point

For the analyses of the distributions of RSS, we collected RSSs in a static setting and applied different post-processing. We recorded a measurement series indoors, from a single access point with a fixed transceiver and access point position. The RSSs were captured in three independent, but temporally consecutive measurement series, each containing about 900 (raw) RSS values. The RSSs of the first measurement series were captured and stored unprocessed. In the second take, RSSs of one second were used to compute their arithmetic mean, which was then stored. The last set of measurements was treated as the former one, but the time period to compute the RSS mean was increased to 5 s; a time interval still practicable when recording fingerprints for an RSS reference database. We will refer to this measurement series as the Single-AP (access point) data.

### 4.2. Gaussian Process RSS Radio Map Modeling

The experiments took place at the faculty of engineering of the Universidad Autónoma de Querétaro (UAQ). The test bed (http://osm.org/go/S9GSwH2Qb?layers=N&m=) includes two, relatively small buildings: both are about 8×45 m; one building has one story; the other has two stories; and the surrounding area is about 80×50 m. These two buildings are divided mostly by soft partitions. The corridor and stairways of the two-story building are semi-open (covered by a roof) and located outside the building; this fact, the dimension of the buildings and the windows and doors being open most of the time give these buildings outdoor properties, with respect to WLAN signal powers.

#### 4.2.1. Radio Map

**Figure 1:**
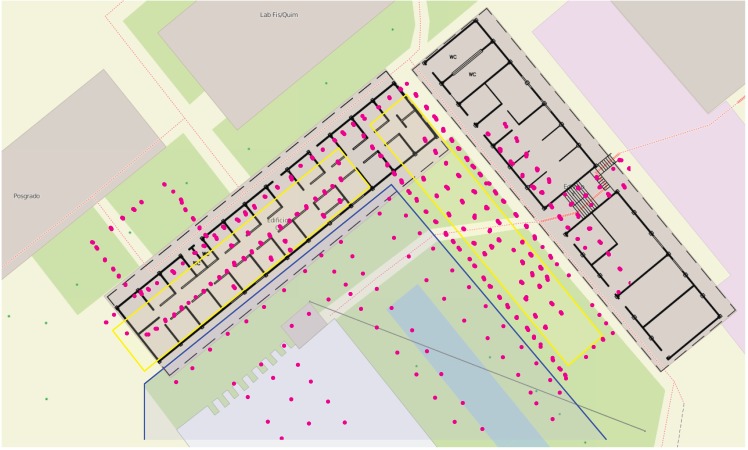
Test area and fingerprint positions (magenta dots) of one access point. Reference points within the yellow shaded area belong to the Indoor dataset; reference points lying in the blue shaded area form the Outdoor-2 dataset; and all other fingerprints belong to Outdoor-1. © OpenStreetMap contributors (www.openstreetmap.org).

The RSS fingerprints were taken in three parts on three different days: one radio map, the Indoor data, contains the fingerprints of the two mentioned buildings, and two other radio maps, called Outdoor-1 and Outdoor-2, contain the fingerprints of the surrounding area. Figure 1 shows the test area. The Indoor data were recorded in the yellow shaded areas and the Outdoor-2 radio map in the blue-framed region, and the remaining fingerprints are collected in the Outdoor-1 dataset.

Each fingerprint contains the mean RSS that was obtained by averaging over about two to five seconds. To account for the experimenter attenuating the signals from a certain orientation, the radio maps Indoor and Outdoor-1 contain up to four fingerprints at each reference position, one for each cardinal orientation.

The reference positions shown in Figure 1 belong to a relatively strong access point, whose signals cover the complete test area. Access points with many fingerprints in the radio map are usually near the test area; hence, their signals are stronger and received more often all over the test area; whereas access points with less fingerprints in the database are supposedly farther away from the test area; therefore their signals are weaker and are received on less occasions. The number of fingerprints from other access points than shown in Figure 1 may be much smaller and possibly situated in a certain region of the test bed. In addition to the three radio maps, we created a fourth one by joining the three radio maps into one: the Test-bed dataset.

#### 4.2.2. Model Fitting and Evaluation

To find the most appropriate Gaussian process prior to predicting an RSS radio map, a variety of mean and covariance functions were evaluated. The squared exponential (SE), the Matérn *Mat*${}_{\nu =1/2[,3/2,5/2]}$ with three different parameters *ν*, the rational quadratic (RQ) and the independent noise kernel (IN) are the kernels we used to construct the different covariance functions. These basis kernels were used directly or they were used in combination with each other, for example as summand (sum of · and ·) or multiplicand (prod of · and ·), to form a new kernel. Furthermore, additive kernels (add of ·) (composed of a sum of low-dimensional kernels) [23] were tested. Moreover, we also included three different mean functions in the evaluation: (1) the zero mean function (Zero); (2) the constant mean function (Const) and (3) the linear mean function (Lin).

The evaluation of the Gaussian process models is based on the test error and the Bayesian information criterion (BIC), both obtained as an average from a 10-fold cross-validation. As test error measuring the root mean square (RMS) of the model residuals was chosen. The BIC is derived from the marginal log-likelihood (see Equation (4)) of the data and contains an additive term penalizing larger numbers of model parameters. Albeit that the data do not meet all of the assumptions for applying the BIC, it yielded a more reliable model measure than using just the marginal log-likelihood of the data; see also [24].

For each of the datasets, one model was trained for each access point; but to assure that the model fitting was able to succeed, we excluded access points with less than 30 fingerprints from the radio maps (in cases with too few fingerprints, the optimization procedure failed).

As each radio map has a different number of access points, a different number of models were trained for each: the Indoor training dataset consists of 49 access points; the Outdoor-1 data has 91 access points; the Outdoor-2 radio map contains 35; and the Test-bed radio map holds 100 distinct access points. (The test area is partially equipped with virtual access points that have distinct MAC addresses. We treated these as distinct access points.)

To fit the Gaussian process models, the gpml package (gpml v3.5, http://www.gaussianprocess.org/gpml/code/matlab/doc/) and GNU Octave (http://www.gnu.org/software/octave/) were used. The hyperparameters for Gaussian process models were found by minimizing the negative logarithmic likelihood function of the training data with a gradient descent method, the limited-memory Broyden–Fletcher–Goldfarb–Shanno (L-BFGS) algorithm. The number of function evaluations of the L-BFGS algorithm was set to 70, and the initial values of the hyperparameters were set to values found by antecedent parameter optimization with a wide range of initial values.

### 4.3. Maximum Likelihood Position Estimation

The localization performance was evaluated with two trajectories along the outdoor area and corridors of the buildings. We refer to these trajectories as Trajectory-1 and Trajectory-2. Both trajectories contain indoor and outdoor sections.

A dataset of a trajectory consists of the RSSs measured at certain positions of the trajectory and the coordinates of this position. To record a trajectory, we used the same software as for the radio maps. This allowed us to record the RSSs and the corresponding positions, which are used as the ground truth. At each position of the trajectories, the RSSs of all receivable access points were captured for about 2–3 s, and their mean was stored and used for further processing.

We used the method described in Section 3 to estimate the position at each point of a trajectory and computed the root mean square of the error of the trajectory as the accuracy measure.

## 5. Results

This section is divided into two parts, according to the two experimental setups in Section 4. The first one presents study results preliminary to the Gaussian process modeling, examining the distributions of RSS raw data and averages. The second part presents the results from the Gaussian process model fitting.

### 5.1. Single-Position Single Access Point RSSs Measurement

In the following, we present the results from the Single-AP data to corroborate the use of averaged RSSs to fit Gaussian process models with the objective of robustly predicting WLAN radio maps.

**Figure 2:**
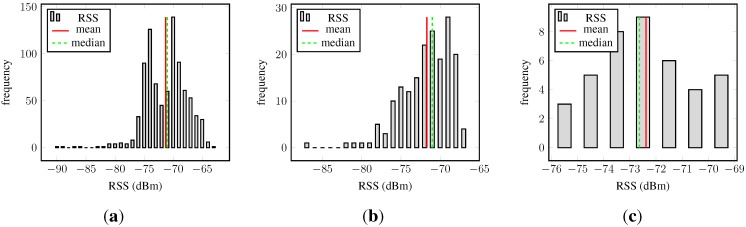
Received signal strength (RSS) histograms at a fixed location: (**a**) Histogram of RSS; (**b**) histogram of RSS averaged over one second; (**c**) histogram of RSS averaged over five seconds. The mean and median are marked by vertical lines.

Figure 2 depicts the histograms of the RSSs, and Figure 2a shows the raw RSS samples.

The histogram is considerably left-skewed and multimodal, showing that these samples are clearly not Gaussian distributed. The skewness is explained by the bounds of the RSS, which was already pointed out in [16].

**Figure 3:**
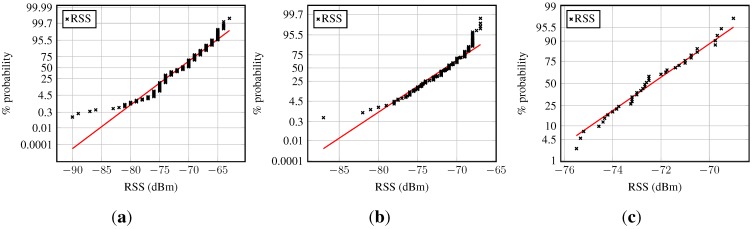
Received signal strength normal probability plot at a fixed location: (**a**) Norm probability plot of RSS; (**b**) norm probability plot of RSS averaged over one second; (**c**) norm probability plot of RSS averaged over five seconds. The linear graph in the normal probability figures connects the first and the third quartile of the data and is then extrapolated towards the ends.

The corresponding normal probability plot, presented in Figure 3a, shows a nonlinear trend, reflects the heavy tail seen in the histogram and confirms the non-Gaussianity of raw RSSs. Furthermore, their discrete nature is visible.

The histogram of the RSS averaged over one second is depicted in Figure 2b, and the normal probability plot of this data can be found in Figure 3b. In both graphs, one may observe that the averaged RSS values are less spread out, bringing the lower and upper bounds closer to the mean. Figure 3b shows that the mean RSSs are smeared around the formerly discrete values. The normal probability plot is reasonably linear around the central tendency measure, but outliers, especially on the left, but also on the right, are present.

Similar observations apply for the third set of data, the RSSs averaged over five seconds. The trend from the raw RSS to the 1-s averages is further amplified for these data. The panels on the right-hand side (Figure 2c and Figure 3c) illustrate the corresponding histogram and the normal probability plot. The histogram is approximately symmetric; the bounds on both ends came closer to the central tendency measure; and the long tails are reduced. The normal probability plot in Figure 3c shows that almost all data points lie on the line indicating normality, hence suggesting a Gaussian model for the RSSs averaged over 5 s. However, the tails are still slightly fat.

In addition, instead the mean and the variance being possibly more consistent and therefore more robust, measures of central tendency and dispersion may be used; especially under non-Gaussian conditions. For instance, the median and the median absolute deviation (MAD) are known to be more robust with respect to outliers from normal distributions. However, only a measure of central tendency is of interest, and since, the difference between the mean and median is small compared to the standard deviations of RSS (see Table 1 and [16]), we prefer the mean over the median because of its more convenient properties.

**Table 1:** Mean and median of static RSS measurements (these values correspond to the mean and median shown in Figure 2a–c).

Central Tendency Measure	Raw RSS	1-s Averaged RSS	5-s Averaged RSS
mean	−71.29	−71.69	−72.38
median	−71.00	−71.00	−72.63

### 5.2. Gaussian Process Model Determination

In order to find the most appropriate Gaussian process prior distribution, different mean and covariance functions were used to train various models with the three radio maps and the dataset that combines these radio maps. We evaluated the models on the basis of the test error and the BIC, averaged over all access points. Additionally, some predicted mean and covariance functions and the residuals (Section 5.2.2) are examined for a few selected access points.

Three different mean functions have been used to fit the model: the zero mean function, the constant mean function and the linear mean function. These mean functions were combined with basis kernels (SE, *Mat* ${}_{\nu =1/2[,3/2,5/2]}$, RQ). The independent noise kernel (IN), which we combined additively with the basis kernels, resulted consequently in a poor model fit (larger residual and BIC), when compared to the model without this kernel, and was therefore excluded from the results. Since the hyperparameter optimization did sometimes not succeed when using an additive kernel with order higher than one, we also withhold these data. Further kernel functions, such as periodic ones, were not examined, because we expect only local structures due to the log-distance path loss model and due to the lower and upper bounds of RSSs.

#### 5.2.1. Test Error and Bayesian Information Criterion

The following tables show the test error and the BIC of the predictions obtained from Gaussian process regression with priors formed by the combinations of mean and covariance functions as shown in the tables. A better fitting model is indicated by the smallest test error and the lowest BIC.

The model measures for the Indoor training data can be found in Table 2.

Consider the test error with respect to the mean functions. One can observe that the constant mean function outperforms the zero mean and the linear mean function consistently; the zero mean function yields always lower test errors than the linear mean function. The BIC shows contrasting results. The linear mean function presents most times a lower BIC than the constant mean function; anyhow, they are very close. The BIC for the models with zero mean function is the largest at all times when compared to the other mean functions.

With regard to the different kernel functions, the test error does not point to a candidate. The model with the additive SE kernel yielded the lowest test error; the SE and Matérn${}_{\nu =1/2}$ kernel show a similar result. The SE kernel works better when combined with the zero mean function and the Matérn kernel better with the constant mean function. The BIC reveals more clearly the more appropriate models: the Matérn${}_{\nu =5/2}$ kernel fits the data best followed by the Matérn${}_{\nu =3/2}$ and then followed by the squared exponential kernel. As expected, the more complex models (the last three) have larger BICs than the models with fewer parameters. Note that the model derived with a zero mean and squared exponential covariance function is also a model with few parameters, but yielding a relatively high BIC.

**Table 2:** Test error and BIC of a Gaussian process regression for different Gaussian process models trained with Indoor data. Model measures are averages over several access points and 10-fold cross-validation. The lowest values are highlighted.

Mean Function/Kernel	Test Error/dBm			BIC		
	Zero	Const	Lin	Zero	Const	Lin
SE	9.856	9.714	9.864	703.093	682.127	682.051
Mat${}_{\nu =1/2}$	9.727	9.686	9.890	691.012	684.433	684.421
Mat${}_{\nu =3/2}$	9.829	9.715	9.916	693.923	682.010	681.903
Mat${}_{\nu =5/2}$	9.847	9.741	9.915	697.181	681.882	**681.730**
RQ	9.796	9.727	9.900	691.367	684.291	684.075
sum of SE and SE	9.860	9.709	9.922	707.760	686.931	687.104
prodof SE and SE	9.900	9.723	9.854	707.089	686.870	686.856
addof SE${}_{0=1}$	9.765	**9.684**	9.762	704.530	693.851	694.259

**Table 3:** Test error and BIC for different Gaussian process models trained with the outdoor data. The values are averages over several access points and 10-fold cross-validation. The lowest test error and BIC are highlighted.

	Mean Function/Kernel	Test Error/dBm			BIC		
		Zero	Const	Lin	Zero	Const	Lin
Outdoor-1	SE	8.982	9.025	9.085	1015.224	994.559	994.662
	Mat${}_{\nu =1/2}$	9.075	9.041	9.090	999.759	993.034	992.891
	Mat${}_{\nu =3/2}$	9.087	9.054	9.101	1004.740	991.610	**991.580**
	Mat${}_{\nu =5/2}$	9.065	9.051	9.097	1009.041	992.243	992.330
	RQ	9.064	9.055	9.107	1002.027	995.405	995.051
	sum of SE and SE	8.984	9.034	9.068	1020.611	999.941	1000.402
	prod of SE and SE	9.008	9.026	9.082	1020.691	1000.822	1000.060
	add of SE${}_{o=1}$	8.971	**8.964**	9.017	1032.788	1021.243	1020.440
Outdoor-2	SE	7.315	7.348	7.401	372.575	356.170	357.956
	Mat${}_{\nu =1/2}$	7.326	7.289	7.333	366.333	357.939	360.296
	Mat${}_{\nu =3/2}$	7.338	7.322	7.382	368.586	356.720	358.833
	Mat${}_{\nu =5/2}$	7.345	7.335	7.393	370.125	**356.221**	358.537
	RQ	7.354	7.361	7.418	367.016	357.939	359.611
	sum of SE and SE	7.317	7.337	7.401	376.122	359.774	361.995
	prod of SE and SE	7.296	7.339	7.386	376.388	359.774	362.188
	add of SE${}_{o=1}$	7.225	**7.207**	7.283	375.852	366.793	367.726

Based on the test error and BIC and contemplating both model measures, we consider a Gaussian prior with constant mean function and Matérn${}_{\nu =5/2}$ covariance function as the best candidate for this dataset.

In Table 3, the test error and the BIC from the outdoor data sets can be observed.

Give consideration to the mean functions first. For both outdoor datasets, mostly the constant mean function provides the lowest test errors, but also models trained with the zero mean function yielded low test errors: in 38% of the models trained with the Outdoor-1 radio map and in 50% of the models fitted to the Outdoor-2 data. Regarding the BIC for the Outdoor-1 radio map, on average, the constant and the linear mean function perform equally well, even though the linear mean function achieved the lowest BIC. For the Outdoor-2 data, the constant mean function provides throughout the lowest BIC. The zero mean function on the contrary produced models that attained always the largest BIC.

Choosing a kernel based solely on the smallest test error would result again in the additive SE kernel function, followed by the SE and the kernels derived as the sum or product of the SE kernel. However, taking the BIC into account shows that exactly these kernels fit the data worst, particularly when combined with the zero mean functions. For both outdoor radio maps, the lowest BICs resulted from models with the Matérn class or squared exponential functions.

We believe a model based on the constant mean function and the Matérn kernel with ν=3/2 or ν=5/2 balances well between the obtained test errors and BICs for the outdoor data. Comparing this to the outcomes from the Indoor data also implies that the Gaussian process models incorporating a Gaussian prior distribution with constant mean and Matérn covariance function fit indoor and outdoor radio maps equally well.

The results from the data joining the three radio maps (see Table 4) confirm the already obtained findings.

**Table 4:** Test error and BIC for different Gaussian process models trained with the Test-bed data. Model measures are averages over several access points and 10-fold cross-validation. The lowest values of each category are typeset in bold.

Mean Function/Kernel	Test Error / dBm			BIC		
	Zero	Const	Lin	Zero	Const	Lin
SE	9.277	9.286	9.403	1498.148	1458.365	1460.108
Mat${}_{\nu =1/2}$	9.351	9.318	9.422	1459.503	**1451.745**	1452.113
Mat${}_{\nu =3/2}$	9.346	9.308	9.435	1472.590	1452.499	1453.363
Mat${}_{\nu =5/2}$	9.347	9.305	9.433	1481.816	1454.384	1455.309
RQ	9.334	9.325	9.428	1462.458	1455.182	1456.116
sum of SE and SE	9.278	9.290	9.388	1504.146	1464.460	1466.045
prod of SE and SE	9.274	9.287	9.400	1504.203	1464.387	1466.445
add of SE${}_{o=1}$	9.059	**9.051**	9.141	1544.440	1531.548	1531.577

With respect to the test error, models using the constant mean function or zero mean function outperform models relying on the linear mean functions. The best test error was obtained with priors based on the additive SE kernel, but the corresponding BIC is also one of the largest. Furthermore, the test error of the model trained with the zero mean and SE covariance function is quite low, but the BICs, which were obtained with the zero mean function, are throughout the largest. Once again, the zero mean and linear mean function perform contrary to the low model measures; the zero mean function has the low test errors, but large BICs and, *vice versa*, the linear mean function. On the contrary the constant mean function yielded consistently low model measures.

Consider the model measure for the different kernels: again, only the combination of constant mean function and Matérn kernels attained low test errors and low BICs. The SE kernel yielded again low test errors, but relatively high BICs, as well, which is not expected for a kernel with few hyperparameters (having the same number of hyperparameters as, for instance, the Matérn kernel). This dataset also reconfirms that models with the additive kernel attain very low test errors.

The evaluation of the test error and BIC for the presented data already provides some insights, which we summarize:

- In all datasets, it can be seen that the lowest BIC was achieved when the constant mean function was combined with the Matérn kernels with the roughness parameter: ν=5/2 for the indoor data, ν=3/2 and ν=5/2 for the two outdoor datasets and ν=1/2 for the joined radio map. The same models yielded low test errors, as well.
- Models employing the zero mean function produced the largest BICs no matter the dataset. As the SE function only result in low test errors when the zero mean function was used, we sort out the squared exponential kernel as an immediate choice.
- For all datasets, the lowest test error was obtained by models with a constant mean function and an additive SE kernel of order one; however, the BIC obtained by these models is much larger than, for instance, the BIC of the models with Matérn functions, indicating a poor fit.
- The test error and BIC obtained from models trained with either indoor or outdoor data are not distinguishable. In other words, for the available data, the choice of the Gaussian process prior distribution does not affect the final model notably.

For the Matérn class functions, we choose ν=3/2 as a tradeoff between general applicability (roughness *versus* smoothness) and model fit (BIC and the test error are in all cases very close to the lowest model measures)

Up to this point, we only narrowed down the search for the most suitable prior distribution to a combination of the constant mean function with a kernel from the Matérn class or with the additive squared exponential function; all of following results are obtained by Gaussian process models based on a prior with a constant mean function.

**Figure 4:**
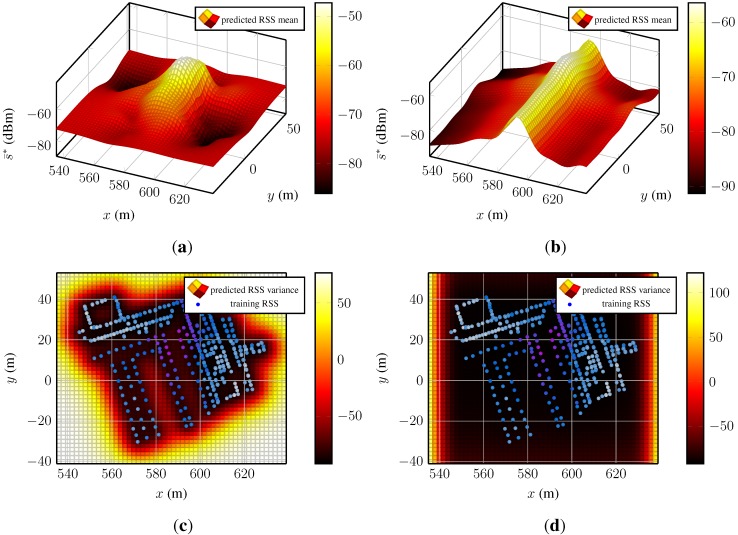
Predicted RSS mean and covariance functions of two Gaussian processes of one access point. The two models were obtained by a Gaussian process regression model with prior distributions chosen according the lowest residuals and BIC: constant mean function in combination with Matérn${}_{\nu =3/2}$ and additive SE kernel. (**a**) Predicted RSS mean function of a Gaussian process using a prior with Matérn${}_{\nu =3/2}$ kernel; (**b**) predicted RSS mean function of a Gaussian process using a prior with additive SE kernel of order one; (**c**) predicted RSS mean function of a Gaussian process using a prior with Matérn${}_{\nu =3/2}$ kernel and the corresponding training data; (**d**) predicted RSS mean function of a Gaussian process a prior with additive SE kernel of order one and the corresponding training data. The two processes where trained with the same data. The magnitudes of the mean and covariance function are indicated with the color bars. The panels depicting the covariance functions present the training data, as well. Large training data are colored purple–dark blue and low RSS light blue–white.

The two leftover prior candidates, constant mean function with either Matérn${}_{\nu =3/2}$ or additive SE${}_{o=1}$, are examined as follows. Figure 4 presents the predictive mean functions for the test bed dataset, the model based on the Matérn${}_{\nu =3/2}$ function on the left and the model based on the additive SE kernel on the right.

Figure 4a shows a spatial RSS distribution with a decaying function with a global maximum and local minima and maxima. The corresponding variance function (Figure 4c) is low where information from training data is available and grows towards regions where no information about the underlying process is available. In contrast, the results in Figure 4b,d are not comprehensible. The predicted mean function in Figure 4b shows an almost constant structure along the y-axis, around 590 m. The covariance function in Figure 4d is flat over a large area and rises towards the edges of the test area. The flat region does not reflect the uncertainty that is expected considering the training points. Such a spatial distribution of signal strengths is nonphysical and unexplainable by the existing environment and/or the models of wave propagation.

The mean and covariance functions have shown that a Gaussian process regression with a prior based on the additive SE kernel fails to fit the data and that the small test error is misleading. As a result, we establish the Matérn kernel-based covariance function for the RSS Gaussian process model as the kernel of choice.

We also like to address a point made in [12]: that a model with a prior distribution based on the SE kernel fits RSSs better when the training data are scarcely distributed than a model with a prior using the Matérn kernel. Therefore, we looked also into the model fit obtained from undersampled training data of a single access point from the Test-bed data; only every forth point of the radio map was used to train the model. We compare models using the constant mean plus squared exponential or Matérn covariance function trained with either the complete or undersampled fingerprints. The data in Table 5 confirm the statement of [12], but only if the training data are very scarce.

**Table 5:** Test error and BIC obtained from eight Gaussian process model fits with constant mean function. For each of the two kernels, the model was fitted either using the complete training data (full) or using only a subset of the training data (undersampled) for both access points. The lowest BIC is typeset in bold.

Training Data/Kernel	BIC-AP_D_		BIC-AP_S_	
	Full	Undersampled	Full	Undersampled
SE	3559.841	939.622	1077.977	**288.091**
Mat${}_{\nu =3/2}$	**3542.781**	**936.257**	**1073.987**	288.178

**Figure 5:**
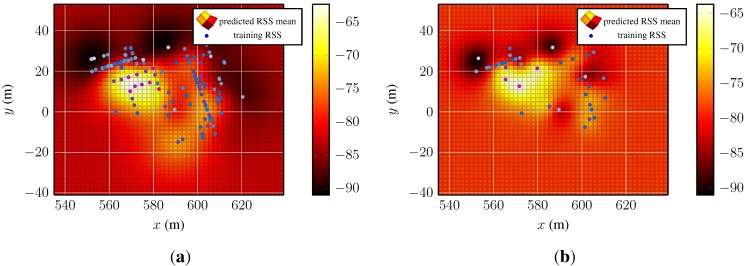
Predictions from two Gaussian process regression models for a single access point. The used Gaussian process prior has constant mean and Matérn${}_{\nu =3/2}$ function. (**a**) Predicted RSS mean of a model that was trained with the full set of its fingerprints; (**b**) predicted RSS mean of a model trained with the fourth part of its fingerprints. The two models were trained with a different number of training data. The magnitude of the mean function is indicated with the color bars. Large training data are colored purple–dark blue and low RSS light blue–white.

The table presents the BICs of eight models, four of these are fitted with data from an access point with a dense fingerprint distribution (AP_D_; see Figure 4c), and four models are trained with data from an access point with a scarce fingerprint distribution (AP_S_, see Figure 5a). If the data from AP_D_ are used to fit the models, the Matérn kernel based model fits the data better than the model with the SE kernel, even if only the forth part of the fingerprints is used. This changes when we use data from AP_S_ to train the models. When using the full set of fingerprints, the Mat${}_{\nu =3/2}$ function-based model outperforms the SE kernel-based model, but if the number of fingerprints is further reduced, the model employing the SE kernel fits the data better.

The reason lies in the greater adaptability of the Matérn function. The Matérn function is more flexible than the smoother squared exponential function; hence, it is able to model spatially very close RSSs, which may vary a lot, better. However, at the same time, it might overfit the data in cases where the structure of the training data is actually smooth.

Nonetheless, the decision when the number of training data is low enough to use the squared exponential function is difficult. Besides the radio map generation process, the principle factors affecting the “visibility” of access points at a certain position are the distance to the access points, objects of the environment and the variance of RSSs. In reality, without additional provisions, the spatial density of RSSs of an access point is hard to control and to predict. The general trend, however, is that the spatial density of fingerprints of an access point is lower in regions far from that access point. Since the Matérn${}_{\nu =3/2}$ function is the more flexible and wider applicable kernel and no indicator of fingerprint density is known, we do not suggest using the squared exponential function.

We conclude this section illustrating the main advantage of using Gaussian process models in fingerprinting techniques compared to basic fingerprinting. Figure 5 depicts the mean and covariance function of the access point and the corresponding training data.

The Gaussian process on the left-hand side (Figure 5a) was optimized with the complete training data and the one on the right-hand side (Figure 5b) with the undersampled training data. Even though only the forth part of the fingerprints was used to fit the model, the main features of the mean function are present: the valleys behind the left building (compare Figure 1) and the peak in [560, 580]×[0, 20]. Only the lack of training samples in [580, 600]×[0, −20] and about (620, 7) in Figure 5a causes the local maximum visible about (592, −14) and parts of the valley behind the right building in Figure 5a to be smoothed over.

#### 5.2.2. Residuals

To see if the chosen Gaussian process is completely able to model the underlying structure of the data, the residuals of the Test-bed data are assessed. We present the residuals obtained from Gaussian process models with constant mean function and Matérn${}_{\nu =3/2}$ kernel, fitted to the RSSs of two access points exemplifying the findings, namely the same access points as in the previous section: AP_D_ and AP_S_; their corresponding fingerprints can be found in Figure 4c and Figure 5a. To illustrate and discuss the residual’s properties, we have chosen scatter plots of the residuals over space, the predictions *versus* training data, and the histogram and the norm probability plot of the residuals. In the following, all panels on the left-hand side correspond to access point AP_D_ and the panels on the right-hand side to the access point AP_S_, respectively.

**Figure 6:**
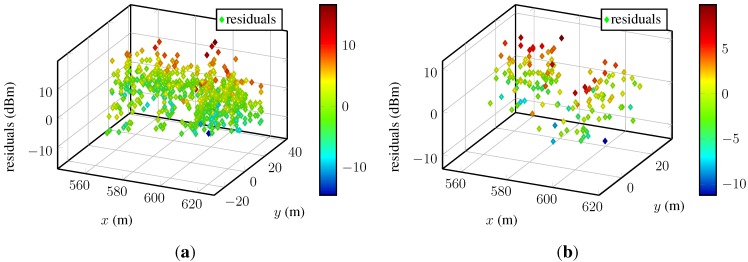
Residuals plotted against space for two different access points: (**a**) An access point with many fingerprints; (**b**) an access point with few fingerprints. The magnitude of the residuals are indicated by the color bars.

To examine the residuals dependent on the fingerprint positions, we refer the reader to Figure 6.

The spatial dependency of the residuals for two-dimensional input data is difficult to assess in these static figures. No perspective allows a proper view, and the evaluation of many different cross-sections would be required to assess the spatial dependency rigorously. Moreover, the unknown positions of access points, possibly affecting the spatial distribution of the residuals, make this even harder. Besides the two examples, we visually examined the residuals of many access points from different datasets. This inspection did not reveal any spatial dependency or systematic pattern of the residuals.

**Figure 7:**
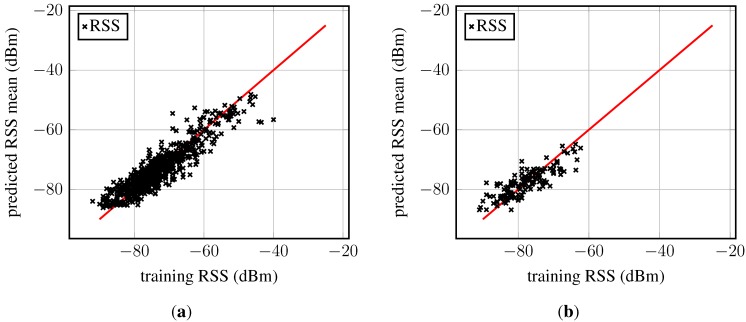
Predicted RSS *versus* training RSS for two access points: (**a**) An access point with many fingerprints; (**b**) an access point with few fingerprints. The red line indicates under- and over-fitting of the training data.

Figure 7 depicts the predicted RSS *versus* the training data. For perfect predictions, all points would lie on the red line; here, the points are basically randomly distributed around that line, indicating an appropriate model fit. However, in both panels (Figure 7a,b), outliers within large training RSS region can be observed, meaning that the largest training samples could not be “reached” by the model’s mean function. This observation suggests that the chosen model underestimates the RSS observations at these peaks (these RSSs are the largest and probably measured very close to the access point). It is noteworthy that not all access points present these outliers. On the lower RSS bound, points concentrate a bit on the upper half in both figures, again suggesting an underestimation. This effect was only observed for a few access points.

**Figure 8:**
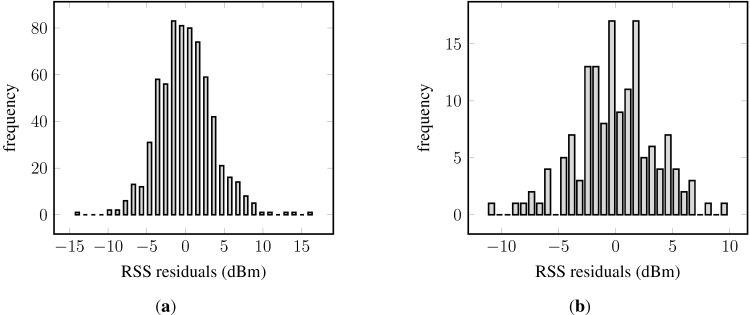
Histogram of residuals of two Gaussian process regression models, each for a different access point: (**a**) An access point with many fingerprints; (**b**) an access point with few fingerprints.

Recall Equation (1); Gaussian process regression expects a Gaussian process prior distribution. Given that a Gaussian distributions was chosen as the likelihood function, the residuals are expected to be about normally distributed and centered around zero. The histograms of the residuals are shown in Figure 8. Most importantly, in both cases, the residuals are symmetrically distributed around zero, though the residuals are not normally distributed. The left-hand side panel shows outliers at the upper and lower bound of the residuals. The histogram obtained from data of AP_S_ (Figure 8b) presents multiple modes, and also, the tails are too large for a normal distribution; however, the small sample size might contribute to these observations.

**Figure 9:**
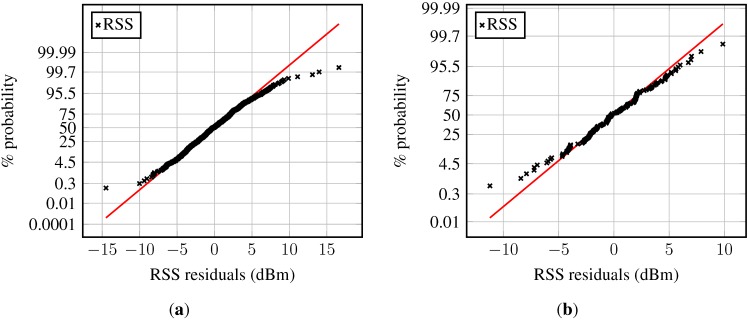
Norm probability plot of the model residuals from Gaussian process regression models of two different access points: (**a**) An access point with many fingerprints; (**b**) an access point with few fingerprints.

For further details, the norm probability plot is examined; see Figure 9 (samples from a normal distribution would ideally lie on the red line). The majority of the residuals in Figure 9a follow a normal distribution. However, heavy tails can be observed, and also, the number of outliers is fairly small: about 1.5% of the sample. The residuals in the second example are approximately Gaussian distributed, as can be seen in Figure 9b. Their distribution also has slightly larger tails than normally-distributed samples would have. The few atypical residuals that exist are not that large, as in Figure 9a.

### 5.3. Positioning Accuracy

The findings favoring the Gaussian process model based on the Matérn kernel were all concluded from the model measures. In the following, we present the results on the positioning accuracy obtained from ML estimators in accordance with Equation (5).

Table 6 contrasts the positioning accuracy yielded by ML estimators derived from Gaussian process models using different covariance functions. All models have the constant mean function in common. The accuracy of the estimators is expressed in terms of the root mean square error and is presented for two trajectories.

The accuracies for both trajectories exhibit the same tendencies: the most accurate Gaussian process model is the one with the Matérn kernel and ν=1/2; the same model that yielded the lowest BIC for the Test-bed radio map; see the corresponding Table 4. The second best model is based on a rational quadratic kernel function, followed by the Matérn${}_{\nu =3/2}$ function. Models using the squared exponential or one of the more complex kernels yielded position errors about 40% larger than that of the best ML estimator. Focusing on the results achieved by the ML estimator with the Matérn kernel and the estimator based on the squared exponential kernel, one can observe an improvement in favor of our proposed model of 1.3 m, in case of Trajectory-1, and 0.78 m for Trajectory-2.

These results on the positioning performance recommend the use of rough kernels over smoother ones. They further demonstrate that the differences seen in the model measure propagate via the likelihood function to the localization error.

The superiority of the Matérn class functions over the squared exponential kernel with respect to WLAN RSS modeling and prediction is hereby confirmed again. However, our proposed Gaussian process model did not perform the best, but the Matérn kernel parametrized with ν=1/2 did.

**Table 6:** Root mean square position error of different position estimators, using the joint radio map, for Trajectory-1 and Trajectory-2. The Gaussian process ML estimators were derived from the Test-bed radio map.

Gaussian Process ML Estimator	Position Error (m)	
	Trajectory-1	Trajectory-2
SE	10.21	6.46
Mat${}_{\nu =1/2}$	**5.90**	**3.97**
Mat${}_{\nu =3/2}$	8.90	5.68
Mat${}_{\nu =5/2}$	8.98	5.92
RQ	6.18	5.30
sum of SE and SE	10.48	6.46
prod of SE and SE	10.46	6.35
add of SE	14.42	11.45

**Figure 10:**
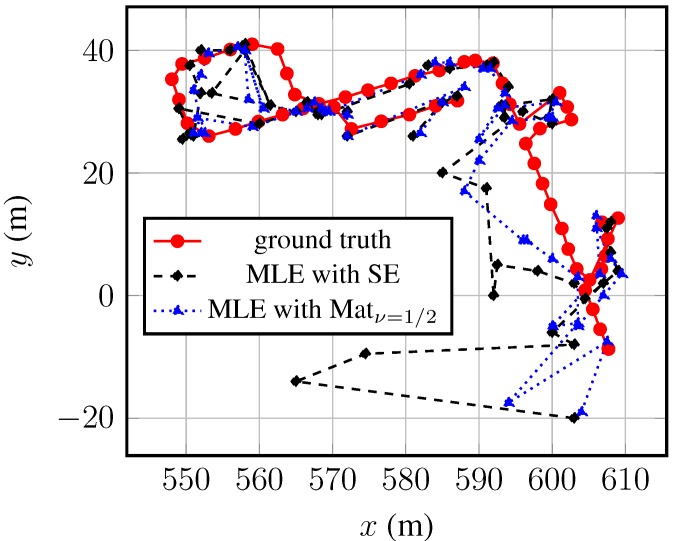
Estimates of Trajectory-1 and the ground truth. The MLEs are based on a Gaussian process model with a constant mean and (1) the squared exponential (SE) or (2) the Matérn class (Mat${}_{\nu =1/2}$) kernel.

Figure 10 depicts *x* over *y* of Trajectory-1 (compare to Figure 1 to relate the trajectory within the test area).

Apparent in this figure are large outliers at its beginning at (607, −9), which are shared by both estimators. From there, the experimenter followed the outdoor corridor and entered the right building two times at [600, 0] × [595, 20] and [603, 27] × [610, 33]. The entering of the building was well estimated, but the section of the outdoor corridor in between presents again large errors. These errors are due to the similarity of the fingerprints, common in outdoor areas. Nothing attenuates the RSSs between the outdoor corridor (left of the indoor area) and the Outdoor-2 area; thus, neighboring fingerprints constitute similar RSSs, which pulls the estimates outward, into the Outdoor-2 area. The same effect can be observed about [550, 25] × [565, 41], a small, empty lawn area. The larger errors in this area are again due to similar neighboring fingerprints. Besides these three problematic sections, typical for location fingerprinting systems, the estimated trajectory follows the ground truth well.

## 6. Discussion

### 6.1. Normality of Averaged RSSs

According to the central limit theorem, the mean of a random variable with finite variance is asymptotically normally distributed. Furthermore, RSSs obey the central limit theorem, as shown in Section 5.1. The RSS averages converge to a normal distribution. Although the RSSs averaged over one and five seconds may not follow a Gaussian distribution, they are more likely symmetric and less likely heavy-tailed than the raw data. Moreover, Kaemarungsi *et al.* [16] argued that symmetric signal strength distributions can be approximated by the log-normal distribution.

On these grounds, we consider that the distribution of RSSs averages resembles a Gaussian distribution sufficiently to justify a normally-distributed likelihood function for Gaussian process regression and to expect more robust predictions as compared to raw RSSs.

### 6.2. Structure Search in RSSs

The model training has to cope with a wide range of conditions: for instance, the number of training points and their spatial density, the contribution of access points to the fingerprints. Furthermore, the dynamics of RSSs is a challenge for the model fitting, especially RSSs of close by access points having a large variance, particularly if the fingerprint density is high, as for example, for the radio maps that contain four fingerprints at almost the same position (recall the four orientations of RSS recording; see Section 4.2.1).

The first part of the Gaussian process modeling is to find an adequate prior distribution, that is its mean and covariance function. The comparison of various combinations of mean functions and covariance functions could narrow down the search to the constant mean function and, regarding the covariance functions, to the Matérn and the additive squared exponential kernel.

We already touched the issue of using the zero mean function to model spatial RSS distributions in Section 1. Gaussian process priors with constant or linear mean function fits RSS data better, because they avoid either that the predictive distribution eventually converges to zero in the absence of training data or an additional estimation step, as in [6,8]. Using the linear mean function is intuitive, because it approximates the decay of RSS over distance and most likely fits the data well when the access point position is at an edge of the test area. On the other hand, if the access point is located in the center of the test area, a linear function only approximates one slope of the RSS reasonably well, but the others respectively poorly; whereas the constant mean function solely assumes that the process converges to a constant value. For the majority of access points in this study, the mean function converged to RSS levels about −80 dBm in regions without training data. A further advantage of the constant mean function is that it has one hyperparameter less than the linear mean function, making the model less complex and the hyperparameter optimization faster. Thus, we recommend the constant mean function as the most appropriate mean function to model WLAN RSS.

The outcome of covariance functions is not surprising given that both kernels are very flexible, able to model smooth, but also rough changes in WLAN RSS. The properties of these two kernel functions explain this result:

To construct an additive kernel, a basis kernel is specified for each input dimension, and the additive kernel becomes a sum of all possible combinations of the input dimensions. The interaction between the different input dimensions take place corresponding to the degree of additive structure defined by the order of the additive kernel. This combinatory capability permits the resulting Gaussian processes to be more flexible than others. Nonetheless, as the hyperparameter optimization for additive kernels of second order was faulty, we used only the first order additive kernel, possibly explaining the structure visible in the right-hand side of Figure 4.

For certain parameters, the Matérn class functions become a product of an exponential and a polynomial function, giving the kernel its adaptability. Furthermore, the roughness parameter contributes to that adaptability: choosing ν=1/2 makes the Matérn function very rough, and for ν→∞, it converges to the SE kernel, which is a very smooth one (it is infinitely differentiable).

The outcome of the positioning experiment favors rough kernels. If the radio map is very dense, the Matérn kernel with ν=1/2 or even the rational quadratic kernel might be more appropriate. As we use Gaussian processes with the objective of interpolating radio maps, dense radio maps are rather the exception. In light of the findings of the positioning experiments, we still recommend the Matérn${}_{\nu =3/2}$ kernel as the basis for a Gaussian prior covariance function.

Given that the combination with the independent noise kernel, sums and products of kernels and the additive kernel did not fit the data as well as less complex kernels, this suggests that spatial WLAN RSS distributions do not constitute a very complex structure. A simple covariance function based on an exponential function, as the Matérn${}_{\nu =1/2}$ or SE the kernel, is able to capture the fundamental RSS patterns. Recall the tables of Section 5.2.1 and focus on the kernels that yielded the lower BICs (SE, Mat, RQ), then it becomes evident that not choosing the zero mean function improves the model more than the choice of the covariance functions.

This study did not disclose a Gaussian process model particularly for indoors or outdoors, refuting our hypothesis. However, the Gaussian processes that model RSSs indoors or outdoors might still have considerably different structures due to the hyperparameters, which were optimized with respect to the data; but an examination of the hyperparameters for six randomly chosen access points and all datasets did not support our hypothesis either; the hyperparameters lacked any pattern.

We identified several possible reasons why the RSS characteristics of the Gaussian process models did not differ between the indoor and outdoor environments of our test area: (i) The spatial distribution of RSSs indoors and outdoors is indeed indistinguishable, due to the properties of the buildings, such as size (at least in one dimension), materials (e.g., soft partitions), structure and contained objects; (ii) The discrete fingerprints capture only a small fraction of the true signal power distribution (see Figure 1), and the information we look for is not contained in our datasets; (iii) The fingerprints may capture the information, but the spatial distribution and resolution of fingerprints, which influences the model fitting, outweigh the effect we were seeking; (iv) The tested Gaussian process models are insensitive to the possibly existing differences in RSSs between indoor and outdoor environments, and they smooth out these differences.

A combination of these reasons may actually occur simultaneously.

### 6.3. Quality of Gaussian Process Model Fit

Even though several research groups used Gaussian processes to model and interpolate RSSs, the quality of the fit of these models was never demonstrated. Section 5.2.2 showed that the residuals are indeed random and do not posses a systematic trend.

The lack of structure in the residuals confirms that the Gaussian processes are appropriate to interpolate WLAN RSS accurately.

For RSS distributions with very large dynamics, we found that the model underestimates the peak values. The decision to capture four fingerprints at virtually the same position, but in the four cardinal directions, may contribute to this effect. It eventually generates very high RSS differences over very small distances and forces the Gaussian process mean function to be very rough. However, if the rest of the training data is rather smooth, the model is not rough enough to cover these RSS differences.

Furthermore, we found that the residuals are approximately normally distributed, but often posses heavy tails; as do the distributions of the RSS measurements. To mitigate this issue, one could, on the one hand, extend the duration when the RSSs are recorded and averaged, such that they become more likely normally distributed. On the other hand, one could use a more robust Gaussian process likelihood function, for example a Student’s *t*-distribution or the Laplace distribution. This may decrease the residuals of the Gaussian process models, hence increasing their accuracy and potentially increasing the positioning results, as well.

Besides the fat-tailed distributions, we noticed a few residual distributions that are skewed or have an unrecognizable shape. We found that these distributions belong to access points featuring low power RSSs and that their shape is due to the small RSS sample size and the often discrete RSSs.

We give a last remark with respect to [12]. Based on the synthetic data and the positioning accuracy as a measure of model fit, the authors favored the squared exponential kernel over the Matérn and rational quadratic kernel. Given their data, this is a reasonable choice. However, much more details than just the spatial density of the fingerprints affect the choice of the Gaussian process prior, and again, the effect of the Gaussian process prior’s mean function should not be underestimated.

## 7. Conclusions

Gaussian processes are indeed appropriate to model RSS for WLAN location fingerprinting. Relatively simple models are able to model the spatial structure of RSS entirely.

The very common use of the “default”, zero mean/squared exponential kernel, model in the field of WLAN fingerprinting is reasonable, but better model fits can be achieved with different mean and covariance functions. The most suited and general Gaussian process regression model has a Gaussian process prior with constant mean function and Matérn class covariance function with ν=3/2. The superiority of the Matérn kernel over the squared exponential kernel was confirmed by the results of the positioning accuracy, achieved by their ML estimators. Our proposed model is proficient for indoor and outdoor environments. Different model fits for indoor and outdoor environments could not be discovered.

The robustness and versatility of Gaussian processes allow the use of a common model to approximate spatial RSS distributions, even with observations that violate the model’s assumptions; the use of averaged RSSs contributes to this versatility and should be preferred over RSS raw data. Such a general model actually facilitates its further application, as particular models for certain areas are unnecessary.

In the first instance, these models help to reduce the effort of radio map creation, compared to classical fingerprinting, while increasing the spatial resolution of RSS potentially improves the location accuracy. What is more, Gaussian process mean functions provide a basis for continuous likelihood functions that can be used for Bayesian location finding and data fusion, wherefore the predicted covariance function is a measure of uncertainty.

A more profound assessment of the influence of different Gaussian process models on the positioning accuracy is an open task.

## Supplementary Material

The three radio maps (Indoor, Outdoor-1, Outdoor-2) are published with this article. They are provided in SQLite 3.x database format as supplementary material, named File S1, indoor.db, File S2, outdoor-1.db, and File S3, outdoor-2.db.

The optimized hyperparameters of six access points, mentioned in the discussion in Section 6.2, are also included as supplementary material and are contained in Table S, hypertable.pdf.
