# Supplementary material for: Revisiting Gaussian Process Regression Modeling for Localization in Wireless Sensor Networks
Source: Sensors (Basel). 2015 Sep 8;15(9):22587–615. doi: 10.3390/s150922587 (PMC4610506; doi:10.3390/s150922587)
Supplement: Supplementary file 1 [file sensors-15-22587-s001.zip › Supplementary Information/hypertable.pdf]

OPEN ACCESS

sensors

ISSN 1424-8220

www.mdpi.com/journal/sensors

Supplementary Materials

Revisiting Gaussian Process Regression Modeling for Localization in Wireless Sensor Networks. *Sensors* 2015, *15*, 22587-22615

Philipp Richter * and Manuel Toledano-Ayala

Facultad de Ingeniería, Universidad Autónoma de Querétaro, Cerro de las Campanas s/n., Col. Las Campanas, Santiago de Querétaro 76010, México; E-Mail: toledano@uaq.mx

* Author to whom correspondence should be addressed; E-Mail: philipp.richter@uaq.mx; Tel.: +52-192-1200-6023; Fax: +52-192-1200-6006.

The following tables contain the optimized hyperparameters of the covariance function of Gaussian process regression models fitted to data of three environments. Each table shows the hyperparameters for six models corresponding the six access points AP 1–AP 6. The Gaussian process prior distribution of that model is specified by a constant mean function and Matérn class functions with roughness parameter $v = 1/2$ (Table S1), $v = 3/2$ (Table S2) and $v = 5/2$ (Table S3).

Table S1. Hyperparameters (ℓ—length scale, σ_f—signal standard deviation, σ_n—noise standard deviation) optimized with RSS training data from different regions (indoor, outdoor-1, outdoor-2) of Matérn covariance function with $v = 1/2$.

	**Indoor**			**Outdoor-1**			**Outdoor-2**		
	ℓ	σ_f	σ_n	ℓ	σ_f	σ_n	ℓ	σ_f	σ_n
AP 1	34.4	8.2	3.7	55.3	13.6	3.5	27.6	8.4	3.8
AP 2	19.5	6.5	3.4	29.8	11.2	3.5	53.6	10.4	2.8
AP 3	16.9	14.5	5.3	31.0	8.4	3.7	13.4	6.1	4.8
AP 4	13.0	13.8	4.8	75.0	6.1	3.5	11.9	9.2	0.0
AP 5	29.9	11.1	4.4	19.0	7.4	3.6	23.0	5.1	0.9
AP 6	14.0	2.4	2.9	4.4	4.2	3.6	9.3	3.3	3.8

Table S2. Hyperparameters (ℓ—length scale, σ_f—signal standard deviation, σ_n—noise standard deviation) optimized with RSS training data from different regions (indoor, outdoor-1, outdoor-2) of Matérn covariance function with $v = 3/2$.

	**Indoor**			**Outdoor-1**			**Outdoor-2**		
	ℓ	σ_f	σ_n	ℓ	σ_f	σ_n	ℓ	σ_f	σ_n
AP 1	18.1	9.7	3.8	18.6	14.5	3.8	17.8	7.8	4.5
AP 2	10.1	6.7	3.5	13.2	8.0	4.0	25.6	9.5	3.7
AP 3	7.4	14.6	5.5	13.6	10.0	4.1	13.0	5.7	5.3
AP 4	6.5	13.9	5.2	56.0	8.3	3.7	7.2	9.0	0.6
AP 5	15.7	12.0	4.5	8.6	6.9	3.8	16.1	4.2	2.8
AP 6	10.7	2.7	3.0	3.1	4.0	3.7	9.9	3.1	4.0

Table S3. Hyperparameters (ℓ—length scale, σ_f—signal standard deviation, σ_n—noise standard deviation) optimized with RSS training data from different regions (indoor, outdoor-1, outdoor-2) of Matérn covariance function with $v = 5/2$.

	**Indoor**			**Outdoor-1**			**Outdoor-2**		
	ℓ	σ_f	σ_n	ℓ	σ_f	σ_n	ℓ	σ_f	σ_n
AP 1	14.8	9.5	3.9	14.7	14.2	3.9	15.8	7.6	4.6
AP 2	7.8	6.4	3.5	10.8	7.6	4.0	21.5	9.2	3.8
AP 3	6.1	14.7	5.5	10.4	9.3	4.1	12.5	5.7	5.3
AP 4	5.4	13.9	5.2	44.8	8.1	3.7	6.8	8.6	0.7
AP 5	12.9	11.7	4.5	7.2	6.7	3.8	26.1	5.5	2.9
AP 6	11.6	2.7	3.0	2.9	4.0	3.8	19.1	3.1	4.1

© 2015 by the authors; licensee MDPI, Basel, Switzerland. This article is an open access article distributed under the terms and conditions of the Creative Commons Attribution license (http://creativecommons.org/licenses/by/4.0/).
